# Effects of arteriovenous fistulas and central venous catheters on the cardiac function and prognosis of patients on maintenance hemodialysis

**DOI:** 10.12669/pjms.39.3.7151

**Published:** 2023

**Authors:** Xufeng He, Yang Liu

**Affiliations:** 1 Xufeng He Nanhua Hospital, University of South China, Health School of Nuclear Industry, Hengyang 421002, Hunan Province, P.R. China; 2 Yang Liu, Department of Hemodialysis Room, Nanhua hospital, University of South China, Hengyang 421002, Hunan Province, P.R. China

**Keywords:** Arteriovenous fistula, Central venous catheter, Maintenance hemodialysis, Vascular access

## Abstract

**Objective:**

To investigate the effects of arteriovenous fistulas (AVFs) and central venous catheters (CVCs) on the left ventricular function (LVF) and prognosis of maintenance hemodialysis (MHD) patients.

**Methods:**

This retrospective cohort study included 270 patients (139 with AVF and 131 with CVC) undergoing dialysis with newly established vascular access in the blood purification center of Nanhua hospital, University of South China, from January 2019 to April 2021. Dialysis efficiencies, LVF indexes, and one-year prognoses were compared.

**Results:**

At six and twelve months after the establishment of vascular access, the mean urea clearances (Kt/V) and urea reduction ratio (URR) between the AVF- and the CVC-group were similar (*P*>0.05). The mean LVF values between the two groups were also similar before the establishment of vascular access (*P*>0.05), but the mean values of left ventricular end diastolic diameter (LVEDd), interventricular septal thickness (IVSTd), and left ventricular posterior wall thickness (LVPWT) in the AVF-group were higher than those in the CVC-group one year later, and mean early (E) and late (A) diastolic mitral velocities, E/A, and ejection fraction (EF) were lower than those in the CVC-group (*P*<0.05). The incidence of left ventricular hypertrophy and systolic dysfunction in the AVF-group was higher than that in the CVC-group (*P*<0.05). The hospitalization rate of AVF-group (23.02%) was lower than that of the CVC-group (49.61%) (*P*<0.05).

**Conclusion:**

Both AVF and CVC can achieve appropriate dialysis effects in MHD patients. AVF has a negative impact on cardiac function while CVC has a high hospitalization rate.

## INTRODUCTION

Maintenance hemodialysis (MHD) is the most commonly used renal replacement therapy for patients with clinical end-stage renal disease, which can effectively prolong the survival time of patients.^1^ Vascular access is a prerequisite for maintenance hemodialysis (MHD) and the establishment of a long-term effective and functional access is important to improve the patient’s prognosis.^2^ Clinical options for permanent vascular access include autologous arteriovenous fistulas (AVFs), tunnel and polyester sleeve catheters, and graft AVFs, of which the first two are more commonly used. The international vascular access guidelines recommend that autologous AVF as a first choice, followed by the tunnel and polyester sleeve catheter if it cannot be established.^3^

However, the two channels provide different advantages^4^,^5^ and both systems carry a risk of complications. The establishment of vascular access frequently results in increased cardiac workload^6^, which may cause abnormal cardiac function. The occurrence of complications can lead to the vascular access failure, insufficient hemodialysis, other complications and reduction of the quality of life of patients.^7^ As far as we know, there are many studies on the survival analysis of AVF or CVC in MHD patients^8^,^9^,^10^, but only few reports attempt to compare cardiac function and prognosis of AVF and CVC catheter. The objective of this retrospective study was to compare the clinical effects and prognoses of two vascular access systems.

## METHODS

Medical records of 270 dialysis patients (138 men and 132 women) with newly established vascular accesses in the blood purification center of Nanhua Hospital, University of South China, from January 2019 to April 2021 were retrospectively reviewed. Patients who received dialysis through AVF and CVC were categorized as the AVF-group (n=139) and the CVC-group (n=131), respectively. Patients aged ≥18 years with complete imaging data and requiring regular hemodialysis for more than 12 months were included in the study. Patients were excluded if they had (1) presence of unrelated severe diseases or severe organ dysfunction (2) presence of malignant tumor (3) history of surgery or trauma within one year after initiating MHD and (4) mental illness.

### Ethical approval:

The study was approved by the Medical Ethics Committee of our institution. (Approval number 2020-KY-81; date 2020-03-11).

### AVF-group:

The AVF was established by end-to-end anastomosis of the forearm cephalic vein and radial artery, and hemodialysis was administered after the fistula matured.

### CVC-group:

Puncture was done at the right internal jugular vein and tunneled dialysis catheter (Covidien, Inc, USA) placement was performed.

### Hemodialysis treatment^11^:

All patients were required to receive regular dialysis. The dialyzer used was FX80 high-flux dialyzer (Fresenius, Germany), with a membrane area of 1.6 m^2^. Bicarbonate was used as the dialysate, and reverse osmosis water was used as the dialysis water. The blood flow was set as follows: 200-300 mL/min, dialysis flow rate: 500 mL/min, three times/week, four hours/time. Anticoagulation therapy with low molecular heparin was applied during dialysis.

The basic information of patients was collected. At six and twelve months after vascular access establishment, 5 ml of peripheral venous blood was collected to check the urea nitrogen level to calculate fractional urea clearance (Kt/V) and urea nitrogen reduction ratio (URR) using the following equations:

Kt/V=1n (urea nitrogen ratio before and after dialysis-0.008 hours per dialysis) + (4-3.5 urea nitrogen ratio before and after dialysis) × ultrafiltration volume per dialysis / body weight after dialysis. URR = 100 × (1-urea concentration before dialysis / urea concentration after dialysis).

The results of color Doppler echocardiography (Philips IE33, USA) before and one year after the vascular access establishment, including LVEDd, IVSTd, LVPWT, E/A and EF values, were obtained. Incidence of adverse cardiac events including left ventricular hypertrophy, left ventricular systolic dysfunction, and left ventricular diastolic dysfunction were recorded. The criteria for left ventricular hypertrophy were as follows: a left ventricular mass index > 125 g/m^2^ for men and >120 g/m^2^ for women.^12^ Left ventricular systolic dysfunction was defined as an ejection fraction (EF) <50.0%,^13^ and a left ventricular diastolic dysfunction as an E/A <1.0.^14^ In addition, the number of hospitalizations of the individuals in the two groups in one year were counted, and the distribution of causes (bleeding, infection and embolism) was analyzed.

### Statistical analysis:

RStudio (R Version 3.4.4, USA) was used to calculate the sample size. To achieve 80% power with an effect size of 0.4 and a two-sided alpha level of 0.05 between the two groups, an estimated 100 patients was needed for each group in the study. We used the SPSS22.0 software to analyze the collected data. Numbers and percentages [n (%)] were used to present non-grade count data, and χ2 test was used to compare the differences between the two groups. Means and standard deviations (*χ̅*±*s*) was used to present measurement data, and t-test was used to compare the differences between the two groups in the case of normal distribution. *P*<0.05 indicated statistical significance.

## RESULTS

We retrospectively analyzed data from 270 patients, including 168 men and 102 women, divided into two groups (AVF-group and the CVC-group) based on the method of vascular access establishment. The patients’ ages ranged from 26 to 75 years, with an average of 55.84±10.91 years. The primary diseases in the patient cohorts included 115 (42.59%) cases of diabetes nephropathy, 29 (10.74%) cases of chronic pyelonephritis, 41 (15.19%) cases of chronic glomerulonephritis and 85 (31.48%) cases of hypertensive nephropathy. There was no significant difference in the basic information between both groups (*P*>0.05) (Table-I).

**Table-I:** Comparison of basic information between the two patient groups.

Group	n	Gender (men/women)	Age (years)	Primary disease (n)			

				Diabetic nephropathy	Chronic pyelonephritis	Chronic glomerulonephritis	Hypertensive nephropathy
AVF-group	139	92/47	55.36±11.37	64 (46.04)	17 (12.23)	19 (13.67)	39 (28.06)
CVC-Group	131	76/55	56.33±10.42	51 (38.93)	12 (9.16)	22 (16.79)	46 (35.11)
*χ^2^*/*t*	-	1.916	0.729	2.893			
*p*	-	0.166	0.467	0.408			

There were no significant differences in the mean Kt/V and URR values between the two groups at 6 months and 12 months after vascular access establishment (*P*>0.05) (Table-II). Before the establishment of vascular access, the LVF values between the two groups were similar (*P*>0.05). However, a year after the procedure, the values of LVEDd, IVSTd and LVPWT increased in the two groups, and were higher in the AVF group (P<0.05). Both E/A and EF decreased, and they were significantly lower in the AVF-group (*P*<0.05) (Table-III).

**Table-II:** Comparison of dialysis adequacy between the two patient groups (*χ̅*±*S*).

Group (*n*)	Kt/V		URR (%)	

	Established for 6 months	Established 12 months	Established for 6 months	Established 12 months
AVF-group (*n*=139)	1.35±0.15	1.41±0.14	69.40±2.77	76.44±3.92
CVC-group (*n*=131)	1.37±0.16	1.40±0.13	69.95±2.58	76.26±3.04
*t*	0.935	0.078	1.688	0.420
*p*	0.351	0.938	0.092	0.675

**Table-III:** Comparison of cardiac function between the two patient groups (*χ̅*±*S*).

Group (*n*)	Time	LVEDd (mm)	IVSTd (mm)	LVPWT (mm)	E/A (%)	EF (%)
AVF-group (n=139)	Before establishment	47.30±3.18	10.42±1.14	10.38±1.33	59.65±13.57	60.23±8.81
	One year after establishment	50.71±3.40^a^	12.79±1.25^a^	11.65±1.52^a^	55.58±14.96^a^	56.67±9.03^a^
CVC-group (*n*=131)	Before establishment	46.68±3.39	10.33±1.18	10.20±1.32	60.84±13.86	60.09±8.56
	One year after establishment	48.28±3.43^ab^	11.63±1.31^a^	10.77±1.41^ab^	59.31±14.20^ab^	58.97±8.74^ab^

***Note:***

a represents the comparison with that before establishment p<0.05; ^b^ indicates that compared with AVF-group p <0.05.

The incidence of left ventricular hypertrophy and systolic dysfunction were higher in the AVF-group compared to the CVC-group (*P*<0.05) (Table-IV). One year after the establishment of the vascular access, we found a lower hospitalization rate in the AVF-group (23.02%) compared to the CVC-group (49.61%) (*P*<0.05) (Table-V).

**Table-IV:** Comparison of adverse cardiac events between the two patient groups [*n* (%)].

Group	*n*	Adverse cardiac event		

		Left ventricular hypertrophy	Left ventricular systolic dysfunction	Left ventricular diastolic dysfunction
AVF-group	139	99 (71.22)	48 (34.53)	79 (56.83)
CVC-group	131	78 (59.54)	25 (19.08)	70 (53.43)
*χ^2^*	-	4.075	8.159	0.315
p	-	0.044	0.004	0.575

**Table-V:** Comparison of hospitalization between the two groups [*n* (%)].

Group	*n*	Reason for hospitalization			Total

		Poor bleeding	Infect	Embolism	
AVF-group	139	0 (0)	11 (7.91)	21 (15.11)	32 (23.02)
CVC-group	131	15 (11.45)	22 (16.79)	28 (21.37)	65 (49.61)
*χ* ^2^	-	-	-	-	29.172
p	-	-	-	-	<0.001

## DISCUSSION

This study compared the dialysis effects of AVF and CVC and their impact on the prognosis of patients undergoing MHD. We found that both methods of vascular access can achieve good dialysis effect, but AVF has greater impact on cardiac function, and CVC is associated with a higher hospitalization rate.

Our study supported the previous studies that have demonstrated the dialysis effects of AVF and CVC in MHD patients.^15^,^16^ We found similar Kt/V and URR values at six and twelve months after the vascular access establishment in the patients of the two groups (*P*>0.05), which suggested that both vascular access systems can achieve appropriate dialysis effects.

Our study also showed that AVF negatively affects cardiac function, as the LVEDd, IVSTd and LVPWT values increased in both groups and were higher in the AVF-group. At the same time, E/A and EF values decreased in both groups and lower in the AVF-group (*P*<0.05). The results of the study are in general agreement with Reddy et al.^17^ In addition, the incidence of adverse cardiac events in patients with AVF was higher than that in patients with CVC (*P*<0.05), suggesting that in patients undergoing MHD, AVF has a stronger negative impact on the cardiac function than CVC, which supported the findings by Faull et al^18^ and Stoumpos et al.^19^ We may speculate that, since 42.59% of the patients in our cohort had diabetes nephropathy and 31.48% had hypertension nephropathy, these conditions may lead to abnormal left ventricular function in both groups.^20^,^21^ Additionally, AVF access adversely affects the heart because of the increased workload required for vascular access blood flow.^22^ Blood passes through the AVF faster than through a typical blood vessel, and the increased blood flow makes the heart pump harder, resulting in long-term stress on the heart that can lead to left ventricular hypertrophy and eventually cause ventricular systolic dysfunction. In contrast, the effect of CVCs on the cardiac load is relatively mild.^23^ Based on our findings, we suggest that in clinical practice, patients undergoing AVF for MHD, especially those with adverse cardiac events, should have their cardiac function monitored regularly to reduce the risk of an adverse prognosis.^24^

MHD treatment is mostly provided in the outpatient department, but many patients require hospitalization due to treatment-related complications. Frequent hospitalizations can seriously reduce the quality of life of patients. Many clinical studies have shown that the most common causes for hospitalization of these patients are cardiovascular and cerebrovascular diseases, infections and access complications.^25^,^26^ In the current study, the hospitalization rate of patients with AVF was lower than that of patients with CVC (*P*<0.05), suggesting that patients undergoing MHD with CVC might have a poor prognosis. Our results are consistent with Almasri et al.^27^ It is plausible that despite optimized biocompatibility, the catheter still carries a high risk of infection and access related complications as a central venous foreign body. In agreement with this assumption, Chiu et al^28^ reported that infection was the main cause of death / hospitalization of CVC patients. As for AVF, it can maintain the complete vascular endothelium, and not interfere with its ability to effectively resist microbial invasion and growth, thus reducing the risk of related complications, and the number of hospitalizations.^29^ Therefore, patients undergoing CVC for MHD would require special attention in terms of potential infection-related problems.

### Limitations:

This was a single center study with data collected for one year only. Further follow-up multi-center studies are needed, with large sample sizes and specific populations (such as elderly patients or patients with anemia).

## CONCLUSION

Both AVF and CVC can achieve appropriate dialysis effects in MHD patients, but AVF has a negative impact on cardiac function while CVC is associated with a high hospitalization rate. In clinical practice, a suitable and ideal vascular access should be chosen in conjunction with the actual conditions of the patient. Cardiac function of the dialysis patients should be specifically monitored to effectively improve the prognosis.

### Authors’ contributions:

**XH:** Conceived and designed the study.

**XH** and **YL:** Collected the data and performed the analysis.

**XH:** was involved in the writing of the manuscript and is responsible for the integrity of the study.

All authors have read and approved the final manuscript.
